# Assessment of Pepper Robot’s Speech Recognition System through the Lens of Machine Learning

**DOI:** 10.3390/biomimetics9070391

**Published:** 2024-06-27

**Authors:** Akshara Pande, Deepti Mishra

**Affiliations:** Educational Technology Laboratory, Intelligent System and Analytics Group, Department of Computer Science (IDI), Norwegian University of Science and Technology, 2815 Gjøvik, Norway; akshara.pande@ntnu.no

**Keywords:** Pepper robot, speech recognition, audio features, evaluation metrics, K-means clustering

## Abstract

Speech comprehension can be challenging due to multiple factors, causing inconvenience for both the speaker and the listener. In such situations, using a humanoid robot, Pepper, can be beneficial as it can display the corresponding text on its screen. However, prior to that, it is essential to carefully assess the accuracy of the audio recordings captured by Pepper. Therefore, in this study, an experiment is conducted with eight participants with the primary objective of examining Pepper’s speech recognition system with the help of audio features such as Mel-Frequency Cepstral Coefficients, spectral centroid, spectral flatness, the Zero-Crossing Rate, pitch, and energy. Furthermore, the K-means algorithm was employed to create clusters based on these features with the aim of selecting the most suitable cluster with the help of the speech-to-text conversion tool Whisper. The selection of the best cluster is accomplished by finding the maximum accuracy data points lying in a cluster. A criterion of discarding data points with values of WER above 0.3 is imposed to achieve this. The findings of this study suggest that a distance of up to one meter from the humanoid robot Pepper is suitable for capturing the best speech recordings. In contrast, age and gender do not influence the accuracy of recorded speech. The proposed system will provide a significant strength in settings where subtitles are required to improve the comprehension of spoken statements.

## 1. Introduction

With the advancement of technology, social robots have become more capable, revolutionizing human lives. They have exceeded their roles and become vital societal partners, including in workplaces and households. These machines are equipped with sensors that help them perceive their environment and make them proficient in social interactions. They can participate in natural and meaningful conversations, understand context, and respond accordingly. This enriched human–robot interaction is transforming various domains, such as healthcare, education, and customer service, where intelligent assistance is required. Speech is one of the fields in which intelligent assistance is highly demanded. However, the complete understanding of speech can be challenging due to various reasons. New language learners face one common problem in that they may struggle to comprehend when native speakers communicate fluently. Additionally, online meetings can sometimes make it hard to thoroughly grasp the speaker’s message. In crowded settings, such as large gatherings, background noise can make it problematic for people to hear one another. Moreover, hearing impairments can also affect voice understanding. Another method of communication could be through texts; however, it can sometimes be difficult to see texts properly due to several reasons, such as long distances and dim-light environments. One potential solution to these issues is to combine speech and subtitles together since only displaying subtitles is not enough due to the possibility of ambiguity in written words. By using this multimodal technique, which combines both speech and text, comprehension can be improved and cognitive burdens can be reduced. In such scenarios, integrating the humanoid robot Pepper can be an effective solution as Pepper has speech recognition capability and a display screen. Social robots are popular for establishing human–robot interaction (HRI) in various domains such as healthcare, education, and the service industry. The purpose of robots in healthcare can be related to the engagement of older persons and identifying their stress [1], as well as for the therapy of autistic children [2]. Abdollahi et al. [1] utilized the social robot Ryan for the emotional recognition of elders and employed two modalities for this purpose: facial expression and speech sentiment. Further, they prepared the robot to respond accordingly and demonstrated that this interaction had a positive effect. The involvement of social robots in the education field could enhance students’ engagement and learning [3,4]. Past studies showed the employment of social robots in the hotel industry for interaction purposes [5] and as receptionists [6]. The speech recognition of social robots plays an important role in establishing proper interaction between humans and robots. Pepper, developed by Softbank Robotics, is an outstanding example of a social robot (https://www.aldebaran.com/en/pepper, (accessed on 20 August 2023)). It is famous for its communicating expertise by comprehending and responding to spoken language. The speech recognition functionality provides various advantages to Pepper, such as voice recognition [7], multilingual support [8], context awareness [9], and speech recording [10]. However, like other speech recognition systems, Pepper’s system also struggles to capture speech accurately due to various factors. Furthermore, Pepper is unable to transcribe spoken speech into text format on its own. Thus, it is crucial to incorporate speech-to-text conversion tools for the clarity and understanding of spoken statements.

Speech-to-text technology is very popular and is utilized in many fields, such as healthcare [11], schools [12], and the service sector [13]. Speech recognition can be beneficial for documentation purposes in healthcare settings [14]. A combination of audio-visual content and speech recognition is an effective way to deliver educational content [15]. The speech recognition system of the Pepper robot cannot perform speech-to-text conversion. As per our previous research [10], the potential solution to this issue is to incorporate the speech-to-text conversion tool Whisper into the Pepper robot. It could be advantageous in educational environments to display the text generated by Whisper on Pepper’s screen. Continuing our previous research, we aim to explore the complexities of Pepper’s speech recognition system more deeply by analyzing audio features. Further, the goal is to create clusters based on these features and select the best cluster by evaluating the Whisper-generated text accuracy. Previous studies showed that a combination of audio features along with machine learning applications provides better performance of speech recognition [16,17,18]. To the best of our knowledge, machine learning incorporated with Pepper’s speech recognition system has not yet been explored. The research questions formulated for the present investigation are as follows:

RQ1: What could be the possible solution to evaluate the performance of Pepper’s speech recognition system when both audio and spatial features are considered?

RQ2: Which spatial features, such as age, gender, and distance from Pepper, affect the performance of the speech recognition system?

In the present study, the objective is to combine various audio and spatial features of the recorded speech to better understand the efficacy of the speech recognition system. We selected audio features such as Mel-Frequency Cepstral Coefficients (MFCCs), pitch, spectral centroid, spectral flatness, energy, and the Zero-Crossing Rate (ZCR) to analyze the recorded speech by the Pepper robot. These features are selected in our study because they play crucial roles in different tasks related to audio processing and may contribute to providing meaningful information, such as spectral patterns, frequency, and loudness, as well as being helpful in forming clusters. MFCCs are commonly utilized as an efficient feature for audio processing as they help in various tasks such as speech recognition [19,20] and speaker identification [21,22]. Furthermore, they are also a useful indicator of the human auditory system [23]. The spectral centroid is another popular method for speech processing [24] and is mostly used for classification purposes such as genre classification [25] and mood classification [26]. The spectral flatness feature offers digital signal processing [27] and primarily determines noise-like characteristics [28]. The Zero-Crossing Rate is one of the important indicators of sound signals, and it is useful for determining the dominant frequency [29]. Furthermore, it is widely applied to various audio-processing domains, including speech/music discrimination [30,31], speech analysis for emotion recognition [32], classification of music genre, and singer identification [33]. Audio feature pitch is a valuable tool for the differentiation of various tones present in tonal languages [34]. Moreover, it can also facilitate the detection of speakers [35] and emotions [36]. Energy is an essential parameter of audio processing, which helps in distinguishing emotions and plays an important role in influencing the efficacy of acoustic models for speech recognition purposes [37]. The effectiveness of these features made them suitable for our study. MFCCs can distinguish various spectral patterns, the spectral centroid can differentiate various types of speech, and the spectral flatness helps in separating background noise. With the help of the ZCR, high-frequency components or noisiness can be identified. Pitch and energy are helpful in detecting variations in sound.

**Figure 1:**
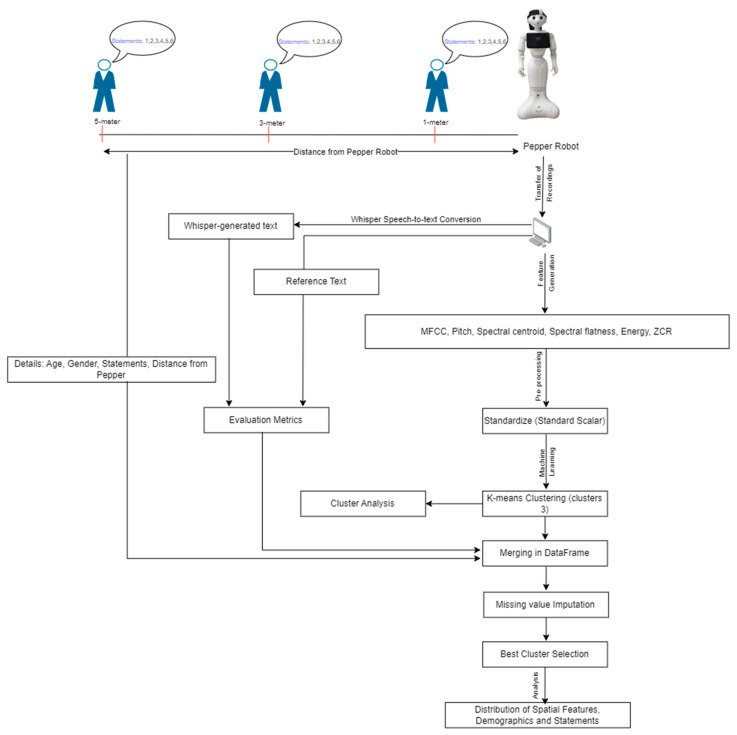
A pipeline of overall work to evaluate the efficiency of Pepper’s speech recognition system.

Furthermore, we aim to investigate how the above-mentioned audio features are related to spatial features such as distance and demographics such as age and gender. Moreover, the goal is to assess Pepper’s speech recognition system performance with the help of clustering audio features and evaluation measures of speech. In order to achieve these goals, the pipeline shown in Figure 1 was followed. In Figure 1, the overview of the experiment and analysis leading to the result has been demonstrated. Firstly, the experiment was conducted to collect the audio data in the form of recordings using Peppers’ sensor. Due to the absence of advanced AI capabilities, Pepper is unable to perform tasks such as speech-to-text conversion and the application of machine learning (ML) algorithms on its own. Hence, these recordings were transferred to the local system, and with the help of Whisper, the corresponding text files for these recordings were generated. In addition to that, audio features were extracted from collected data recordings in the local system. In the next step, K-means clustering machine learning was opted to group features of speech recordings based on their inherent properties. Furthermore, the best cluster can be selected based on the higher accuracy of predicted speech. The structure of the paper is as follows: The literature review for the present study is presented in Section 2. The detailed methodologies are described in Section 3. Section 4 contains the results obtained from this study. Discussions are presented in Section 5. Finally, conclusions are outlined in Section 6.

## 2. Literature Review

One of the main problems usually faced by speech recognition systems is variation in human speech. Accents [38], dialects [39], and speech disorders [40] can influence speech and differences in pronunciation. Different people pronounce the same word in various ways. Feng et al. [41] mentioned that Automatic Speech Recognition (ASR) performance can be influenced by several aspects, such as regional and native accents, age, and gender. Different regions of speakers might have varying speech styles for the same sentence [42]. A person’s age could be another factor in producing differences in speech. Karlsson et al. [43] demonstrated that articulation and phonation can be influenced by age. Bóna [44] showed that age can impact speech style. Das et al. [45] conducted empirical studies examining how various speech characteristics and cepstral features change with aging in the context of Bengali vowels. Kennedy et al. [46] pointed out the limited research on children’s voice recognition and emphasized the need for further investigation in the human–robot interaction domain. Arslan et al. [47] mentioned that speech disfluency increases with aging. However, their findings suggested that gesture along with speech disfluency was comparable for younger and older adults. Previous studies indicated the incapability of Nao and Pepper robots to understand the voices of children [46,48] and elders [49,50]. Another aspect influencing speech is a person’s gender, as there are differences in pitch, frequency, and vocal tract length between males and females. The findings of Mendoza et al. [51] indicated notable distinctions between genders’ breathier quality often observed in female voices. However, Pande et al.’s [52] findings suggest that Pepper’s speech recognition system does not detect much difference between male and female voices. Distance and background noise can also inhibit the precision of speech signals. Increasing the distance from the speech recognition system may impact the recognition accuracy and noise [46]. In noisy surroundings, it is difficult to recognize spoken words correctly [53,54]. This variety creates a considerable barrier for any speech recognition system, including Pepper.

The application of machine learning algorithms on speech data, facilitated by the extraction of meaningful features, carries great importance in various fields, such as emotion recognition [55,56], speaker accent identification [57], and speech disorders [58]. Machine learning algorithms such as K-nearest neighbors [59], Convolutional Neural Networks (CNNs) [60,61], and Deep Neural Networks (DNNs) [62] improve the accuracy of speech recognition. Acoustic features provide insights into sound data. Mel-Frequency Cepstral Coefficients (MFCCs), pitch, spectral centroid, spectral flatness, energy, and the Zero-Crossing Rate (ZCR) are essential features among these features. According to Sandhya et al. [63], spectral features include MFCCs, spectral centroid, spectral flatness, Root Mean Square (RMS), and ZCR. Additionally, the research conducted by Micheyl et al. [64] highlights the connection between pitch and fundamental frequency. 

The MFCC feature plays an essential role in speech recognition [21,22,65]. Gourisaria et al. [66] applied MFCC and Short-Time Fourier Transform (STFT) to extract audio features and achieved higher accuracy with the Artificial Neural Network (ANN) model. Ittichaichareon et al. [20] showed MFCC feature extraction and the utilization of MFCCs to apply a Support Vector Machine (SVM). Hamza et al. [23] demonstrated that with the help of MFCC features and machine learning techniques such as Random Forest, Decision Tree, and SVM, deepfake audio can be identified. Pitch analysis is essential in identifying speakers [35] and emotion detection [36]. Shagi et al. [67] proposed a machine learning approach for gender identification using pitch features of speech. For this purpose, they used ML methods such as a CNN, the Multilayer Perceptron (MLP), an SVM, and Logistic Regression (LR). The spectral centroid is often employed as a measure of sound signal brightness and timbre in music analysis [68], genre classification [25], and mood classification [26]. Ferdoushi et al. [69] demonstrated that the spectral centroid can effectively distinguish heart sounds with a murmur. The spectral flatness is a metric applied to estimate the level of noise, uniformity, and width [70]. Lazaro et al. [71] used an SVM to classify features like the spectral centroid and spectral flatness for music tempo classification. The Zero-Crossing Rate (ZCR) can be applied to classify percussive sounds and separate voice and unvoice [72]. Panda et al. [73] proposed that a multimodal fusion of features such as MFCCs, the Zero-Crossing Rate (ZCR), and Root Mean Square (RMS) with machine learning approaches XGBoost, SVM, Random Forest, Decision Tree, and KNN to increase the accuracy of emotion detection systems. Paul et al. [74] used feature fusions of MFCCs, the ZCR, pitch, and Linear Predictive Coefficients (LPCs) and applied an SVM, the Decision Tree, and Linear Discriminative Analysis (LDA) for emotion detection. Hammoud et al. [75] utilized ZCR, RMS, and MFCC features with various machine learning approaches, including decision tree, random forest, and KNN, and demonstrated that MFCCs with a Random Forest attained maximum accuracy for infant crying interpretation.

In some studies, unsupervised machine learning was applied to speech data [76,77]. Aldarmaki et al. [78] addressed that ASR with labeled data can be challenging, and they reviewed unsupervised ASR along with its limitations. Esfandian et al. [79] employed Gaussian Mixture Models (GMMs) and Weighted K-means (WKM) clustering techniques to effectively reduce the feature space dimensions for speech recognition in the spectro-temporal domain. Hajarolasvadi et al. [80] used K-mean clustering and spectrograms for 3D CNN-based speech recognition. Vyas et al. [81] showed that MFCCs and the K-means algorithm were helpful in detecting mood from Indian music. Bansal et al. [82] applied K-means clustering on MFCCs and Linear Predictive Cepstral Coefficients (LPCCs) to classify emotional Hindi speech. Marupaka et al. [83] utilized MFCCs, ZCR, the Dynamic Energy Ratio (DER), and Cyclostationary features for the classification of sound signals using K-means clustering. Poorna et al. [84] used Hybrid Rule-based K-means clustering and multiclass SVM for emotion recognition and demonstrated that Hybrid-Rule-based K-means clustering performed better than the multiclass SVM. However, no known studies have investigated the use of machine learning algorithms in conjunction with the speech recognition system of the Pepper robot.

Apart from applying machine learning, the other characteristic that can provide ideas about the correctness of recorded speech is speech-to-text technology. Speech-to-text technology has been used in various domains. Some reports focus on speech-to-text utilization in education and learning [85]. Debnath et al. [15] demonstrated that audio-visual content combined with automatic speech recognition can efficiently deliver educational content to individuals with disabilities. In another study, Goss et al. [14] revealed, through a survey, widespread belief in the usefulness of speech recognition technology for clinical documentation while also acknowledging the challenges associated with implementing it. OpenAI (San Francisco, CA, USA) has developed a speech-to-text conversion tool called Whisper [45], which efficiently recognizes speech in several languages and executes numerous tasks successfully. Macháček et al. [86] employed Whisper for the transcription of real-time speech. Vásquez-Correa et al. [87] revealed that with the utilization of synthetic data, Whisper-based Automatic Speech Recognition leads to performance enhancement in certain areas. Spiller et al. [88] noted that incorporating Whisper into audio transcription for mental health research can simplify data analysis.

## 3. Methodology

Experiments were conducted in a controlled closed-room laboratory environment in the Educational Technology Laboratory, NTNU Gjøvik, to test Pepper’s speech recognition system. A laptop with an 11th Gen Intel(R) Core (TM) i5-1145G7 @ 2.60 GHz processor, 16 GB RAM, and running the Windows 10 operating system was used. The experiment was carried out using Python version 2.7.16 and Python version 3.9.13.

### 3.1. Experimental Setup

Eight participants, including three males and five females aged 15–55, were recruited to record six randomly selected statements from Pepper at three distances (1 m, 3 m, and 5 m). The sample included diverse educational backgrounds, including Bachelor’s, Master’s, and Ph.D. degrees. It should be noted that none of the participants spoke English as their first language, and they belong to Asian and European regions. Pepper was used to record statements. There were 18 recordings available for each person, making 144 recordings for eight people.

### 3.2. Pepper Robot Function

In this study, the Pepper robot was used to record and save participants’ speech within its system using the ALAudioRecorder service (http://doc.aldebaran.com/2-5/naoqi/audio/alaudiorecorder.html (accessed on 20 August 2023)). This service allowed the robot to use its microphones to record audio, and the recording process was initiated and terminated using the “startMicrophoneRecording” and “stopMicrophoneRecording” methods, respectively. The “.wav” extension was used to save the audio files.

### 3.3. Transfer of Recordings to the Local System

Paramiko is a Python library that allows secure communication with remote servers through the SSH protocol (https://www.paramiko.org/ (accessed on 20 August 2023)). With Paramiko, an encrypted connection can be established to a remote server and authenticated using a private key or password. It also makes it simple to perform secure file transfers between the Pepper robot and the computer.

### 3.4. Feature Generation

Feature generation plays an important role in analyzing audio recordings. The audio characteristics can be gained through these features, which can further help to extract meaningful information and patterns from the data. In this study, the features were extracted using the Python library ‘Librosa’. The features extracted are outlined in the following subsections.

#### 3.4.1. Mel-Frequency Cepstral Coefficients (MFCCs)

MFCCs are the coefficients that create the Mel-frequency cepstrum (MFC) [65]. The MFC demonstrates the short-term power spectrum of sound. MFCCs can be generated by employing various mathematical operations on the power spectrum of a signal. MFCCs are mainly used for audio and speech processing work, such as speech recognition, the classification of music genres, etc. The set of MFCCs was computed using the ‘librosa.feature.mfcc’ function.

This function takes two input parameters: audio waveform data and the sample rate. The number of MFCCs selected for audio analysis was 13, which shows the number of rows. The number of columns corresponds to the time frames, which were 419. Thus, the MFCC array was set to have a specific shape with 13 rows and 419 columns. Other input parameters, such as frame length and frame shifts, were selected by the function by default.

#### 3.4.2. Pitch

Pitch is used to measure the fundamental frequency of sound [89]. It is mostly used for the recognition of normal speech. The ‘librosa.yin’ function was employed to calculate the pitch of the recorded audio. YIN [90] is a valuable tool for accurately estimating the fundamental frequency of both speech and music signals.

#### 3.4.3. Spectral Centroid

The spectral centroid [91], as the name suggests, is related to the center of mass of the spectrum of sound. Its analysis is related to the texture of a sound. The output of the ‘librosa.feature. spectral_centroid’ function is a set of values that denote the spectral centroid of audio. 

#### 3.4.4. Spectral Flatness

The spectral flatness [27] can be determined by calculating the ratio of the geometric mean to the arithmetic mean of the values within a particular frequency range. It is the feature of noise [92]. A high spectral flatness indicates a uniform power spread, while a low spectral flatness indicates an inconsistent power distribution [70].

#### 3.4.5. Time Domain Features

The Root Mean Square (RMS) and Zero-Crossing Rate (ZCR) are features of the time domain [93]. To calculate the energy present in speech, the Root Mean Square (RMS) value was considered using ‘librosa.feature.rms’. The Zero-Crossing Rate (ZCR) measures how many times a waveform crosses the zero axis within a specific amount of time [94]. Energy and the ZCR are both important components for separating voice–unvoice from sound [95].

### 3.5. Pre-Processing of Dataset

Pre-processing of the dataset is an important step so that the data are clean enough before applying the machine learning algorithm. All features may be present in different scales, and they should be on some common scale. For this purpose, standardization is used. Standardization is a process used on datasets to convert the features into a distribution that resembles a normal curve containing a mean of 0 and a standard deviation of 1. Scikit-learn [96] comes equipped with a module called StandardScaler that carries out data standardization.

### 3.6. Application of Machine Learning

There exist many unsupervised algorithms; however, we selected the K-means algorithm for data analysis because its computational complexity is low and it is efficient as well as simple to implement [97]. Abdalla et al. [98] compared the K-mean algorithm with agglomerative hierarchical clustering (AHC) for small datasets and found that K-means clustering had better performance than AHC for purity and entropy when the cosine similarity measure was applied. However, when the Euclidean distance was employed, AHC performed better than K-means. Rathore et al. [99] showed that in the context of performance, K-means is better suited than hierarchical methods for document clustering. Similarly, Abbas et al. [100] also demonstrated that K-means excels over hierarchical clustering and provides good results for large datasets. 

K-means [101] is an unsupervised algorithm that is data-driven as it identifies clusters based on information present in the dataset. K-means clustering [102] is a well-accepted and widely used [97] unsupervised machine learning algorithm. It is an appropriate selection for acquiring knowledge of the intrinsic properties within data and discovering hidden trends and associations. The K-means clustering algorithm is mostly utilized to organize large datasets [103]. The essential aim of K-means is to divide a dataset into unique groups, or clusters, based on the similar properties of data points within each cluster. By utilizing K-means clustering, it is possible to determine which spatial features belong to the same group. We used the elbow method to select the optimal number of clusters. The elbow method helps in identifying the elbow by plotting the sum of squares (SSEs) against different cluster numbers (k). Further, the K-mean algorithm is fitted on an optimal number of clusters, and the output assigns a cluster label to each data point. The clustering will help in identifying patterns of audio features present in different groups.

### 3.7. Integration with Whisper: Performance Evaluation Using WER, MER, WIL, and CER

Whisper [104] is a versatile open-source speech recognition model that has been trained on a comprehensive audio dataset. Whisper can be used to execute several tasks, including multilingual speech recognition and translation. The audio recordings, which are saved in the Pepper robot, can be transferred to the computer system with the help of the Paramiko library, where Whisper can be used to convert them into text. A Python version, Python 3.9.13, was employed for audio-to-text conversion by Whisper. In Python code, the Whisper library was imported. A pre-trained model called ‘base’ was loaded, which had undergone extensive training on a substantial audio dataset, guaranteeing efficient speech-to-text conversion. Furthermore, the performance of ‘.en’ models is better for ‘base.en’ models [105]. Therefore, we selected the ‘base’ model. This model was applied to convert the recorded audio data into text format using the transcribe(audio_file(in .wav format)) method. The performance of speech recognition tools can be evaluated by evaluation metrics such as WER [106], MER [107], WIL [108], and CER [109]. These evaluation metrics help in comparing the original text and Whisper-generated text. Lower values of WER, MER, WIL, and CER correspond to higher accuracy [110].

### 3.8. Missing Value Imputation

The audio features, evaluation metrics, persons’ demographics (age and gender), statements, and distances (1 m, 3 m, and 5 m) were merged in a pandas dataframe. Further, the missing values for data points were searched using the isnull(audio_file(in .wav format)) function. The missing values were filled with the mean value of the column.

### 3.9. Selection of Best Cluster

The evaluation metrics provide insights into the quality of speech recognition for individual data points. A threshold for Word Error Rate (WER) values was set at less than 0.3 to identify the most suitable data points within a cluster. The cluster with a higher proportion of WER values below 0.3 can be considered optimal.

## 4. Results

### 4.1. Feature Generation from Audio Recordings, Pre-Processing and Machine Learning

**Figure 2:**
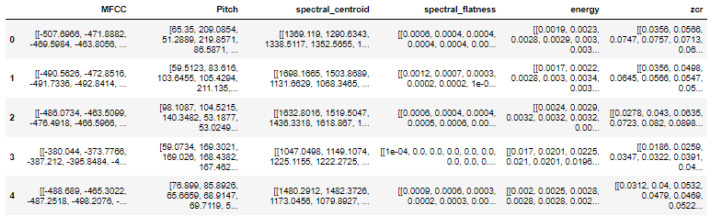
Screenshot of top five rows (out of 18 records) of extracted audio features of a person.

Extracting various features from audio recordings is necessary for feature generation to better understand and analyze the recorded speech. In addition to audio features such as the MFCC, pitch, spectral centroid, spectral flatness, energy, and ZCR, other features such as the distance from the robot, spoken statements, age, and gender may also be required for a comprehensive analysis. Figure 2 shows an overview of extracted audio features from the signal. The figure includes the top five rows and six columns—MFCC, Pitch, spectral_centoid, spectral_flatness, energy, and zcr. There were eighteen recordings for each person, as each person was placed at three different distances from Pepper, and at each position, they spoke six statements. Furthermore, the vector size of each frame was 419. Hence, there were 7542 records generated for a single person. This study recruited eight participants, resulting in a grand total of 60,336 recordings.

**Figure 3:**
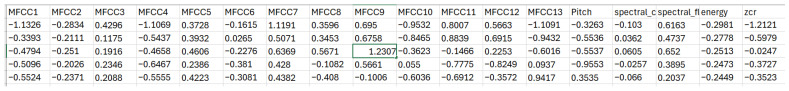
Screenshot of top 5 rows (out of 60,336 records) with standardized 18 audio features.

The features were on different scales, and it is important that they should be on a common scale before applying any machine learning algorithms. StandardScaler was used to obtain the standardized features. Figure 3 contains the top five rows and eighteen columns. The column names from MFCC1 to MFCC13 correspond to thirteen MFCCs. The rest of the columns are for Pitch, spectral_centoid, spectral_flatness, energy, and zcr. It is important to apply machine learning algorithms to determine insights into the audio features.

**Figure 4:**
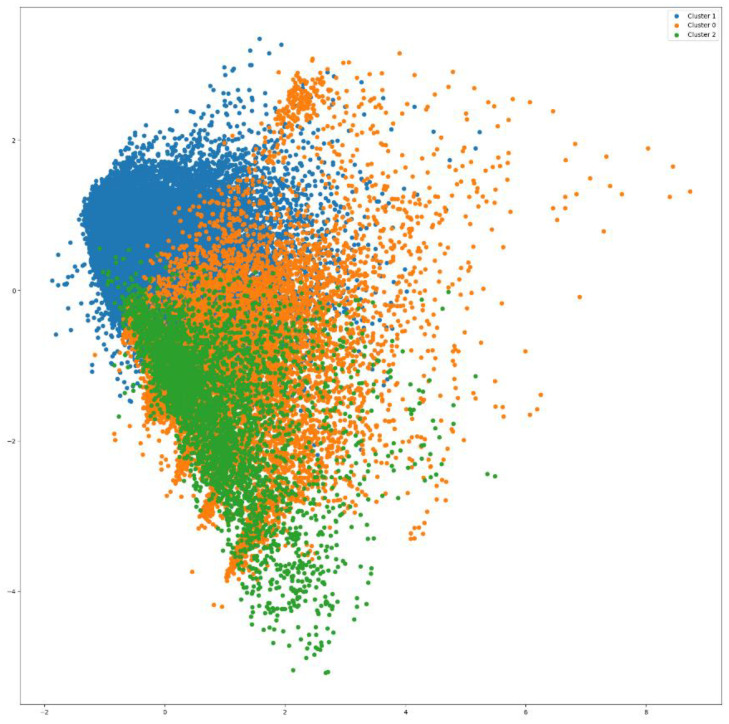
Three clusters generated by K-mean clustering. The color indicators for cluster labels are shown in the box.

K-means clustering was applied here to group the data points based on their feature similarities. Three clusters were formed (Figure 4). As per Figure 4, Cluster 1 is the biggest cluster (indicated by blue color), followed by Cluster 0 (indicated by orange color), and Cluster 2 (indicated by green color) has the minimum number of data points.

### 4.2. Visualization of Clusters with Features

**Figure 5:**
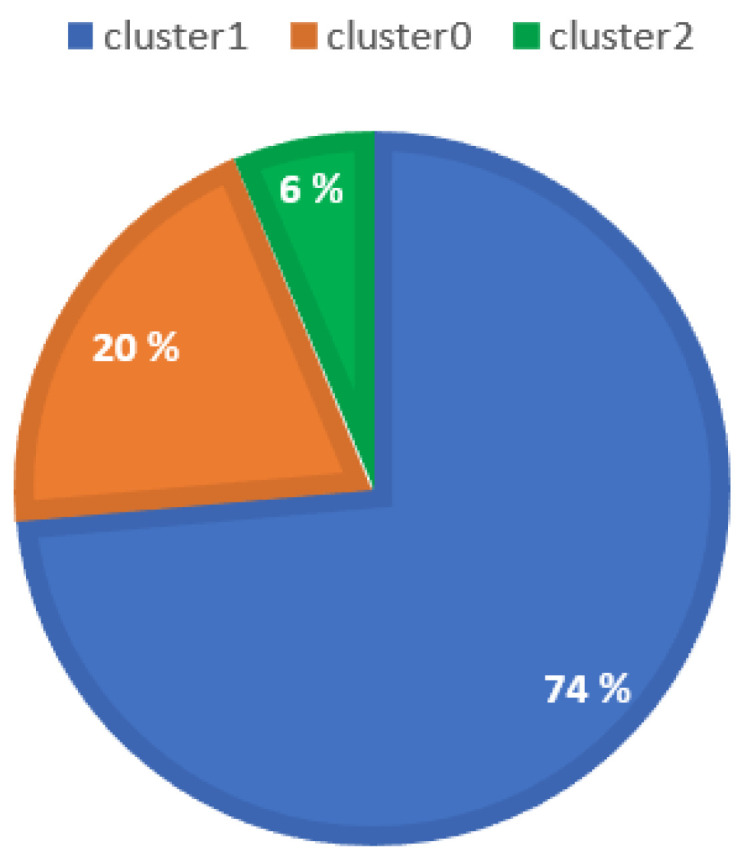
Distribution of data records in each cluster. Cluster 0, Cluster 1, and Cluster 2 are indicated by orange, blue, and green colors, respectively.

The distribution of clusters is shown in Figure 5, which indicates that Cluster 1 has the highest number of records. Cluster 0 is the second-largest cluster, but there is a significant difference in the number of elements compared to Cluster 1. Cluster 2 contains the minimum number of records. It is crucial to visualize the individual features present in each cluster.

**Figure 6:**
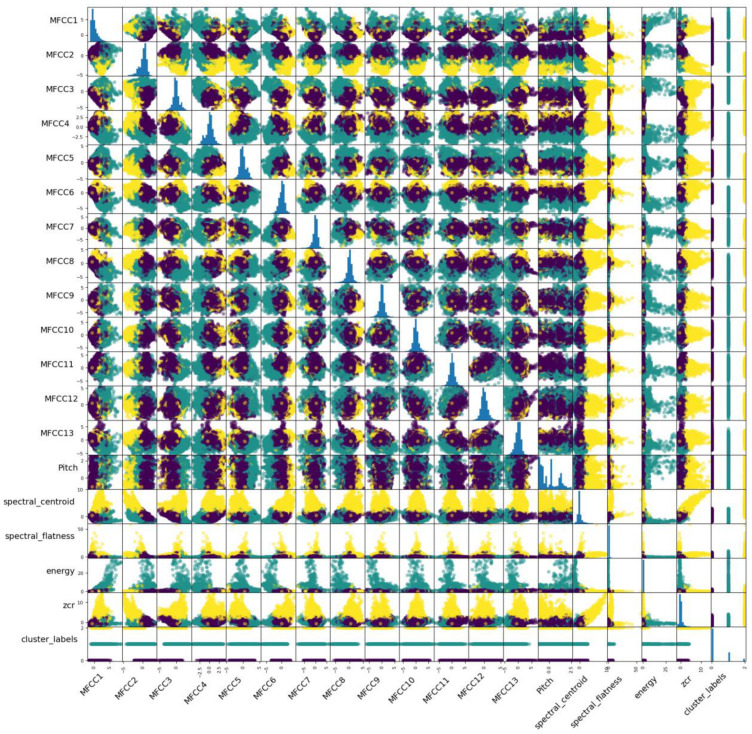
Scatter matrix of all eighteen features in each cluster. Purple color indicates Cluster 0, green indicates Cluster 1, and yellow indicates Cluster 2. Blue color plots in the diagonal represent the data distribution for each feature.

This study utilized a scatter matrix to effectively visualize and analyze a range of audio features and their respective clusters. Figure 6 provides a valuable overview of this matrix, which is particularly helpful in identifying correlations and relationships between feature pairs. Notably, the diagonal of the matrix provides insight into the distribution of individual features within the dataset.

**Figure 7:**
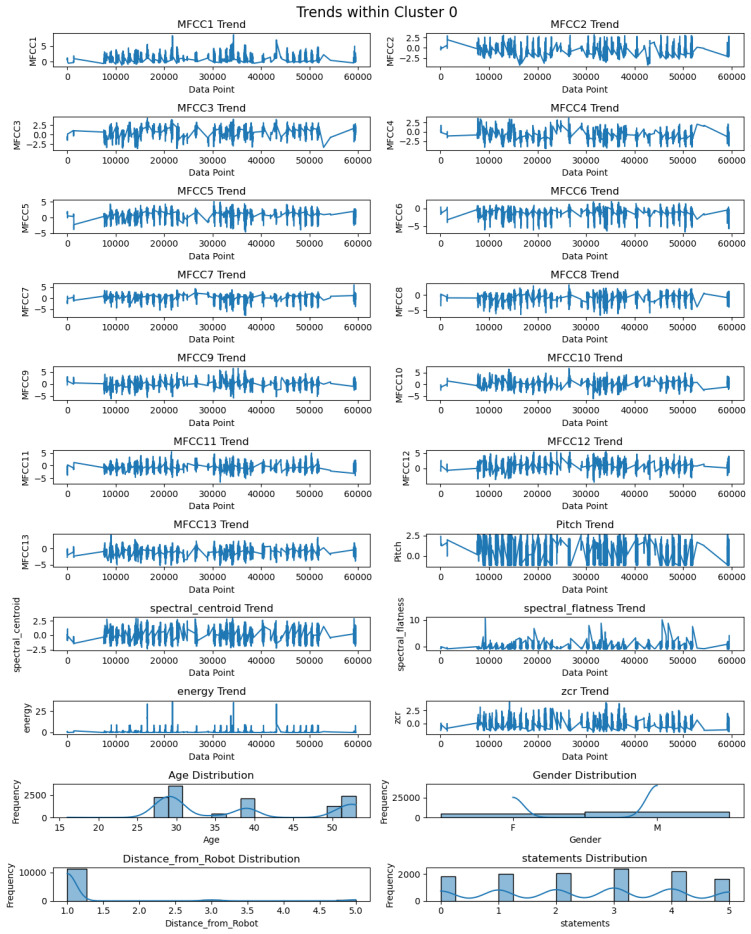
Visualization of trends of twenty-two features in Cluster 0.

**Figure 8:**
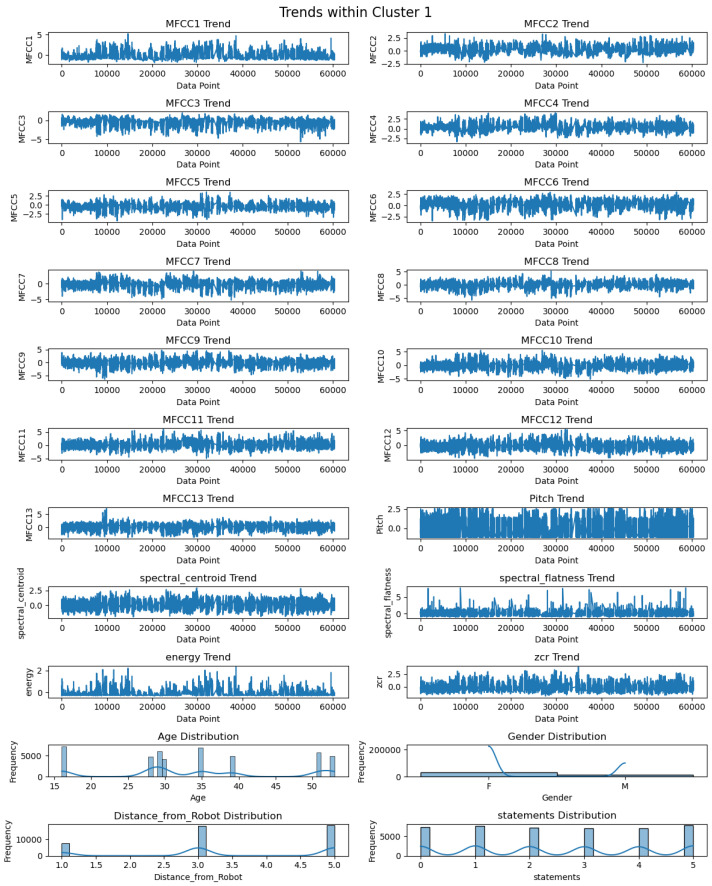
Visualization of trends of twenty-two features in Cluster 1.

**Figure 9:**
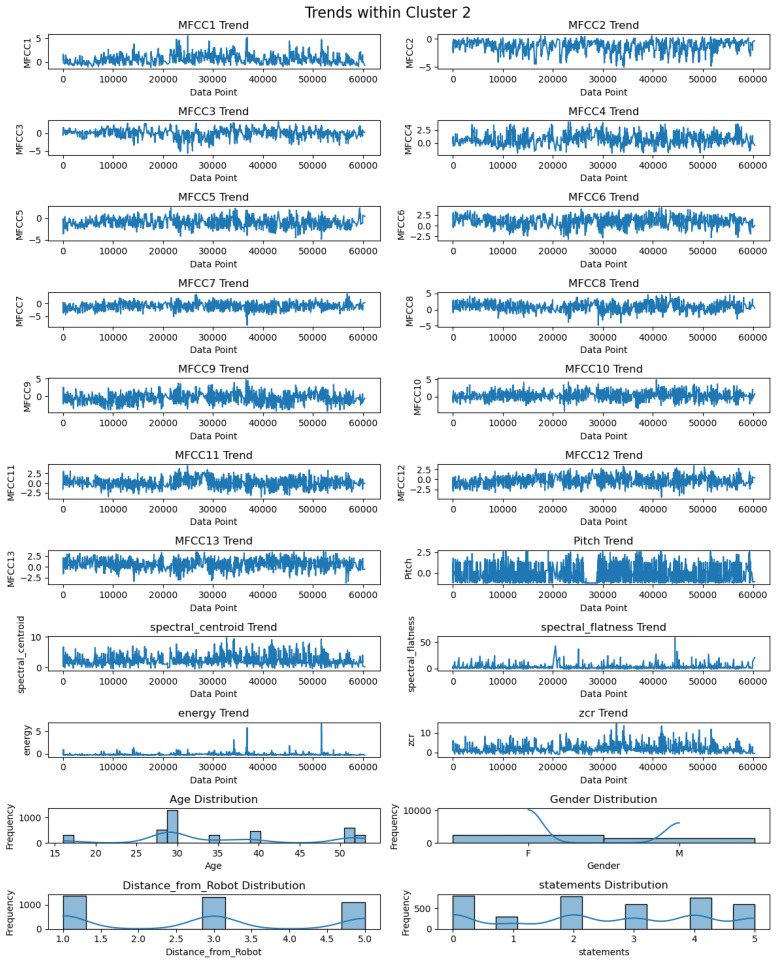
Visualization of trends of twenty-two features in Cluster 2.

In addition, Figure 6 offers an illuminating breakdown of features across different clusters, with purple denoting Cluster 0, green denoting Cluster 1, and yellow denoting Cluster 2. It basically provides information about the pairwise relationships of any of the two features in all three clusters. To further enhance our understanding of these patterns, we have included three separate figures: Figure 7 for Cluster 0, Figure 8 for Cluster 1, and Figure 9 for Cluster 2.

The descriptive statistics of each feature of Cluster 0 are illustrated in Table 1. These descriptive statistics provide a complete view of audio features and contain values for the count, mean, standard deviation (std), min, 25%, 50%, 75%, and max. The central tendency and variability of these features can be computed by observing the mean and standard deviation; however, the range of audio features can be found by observing the corresponding min and max values. As per Table 1, the number of records for each feature in Cluster 0 is 44406. The mean values of MFCCs lie between −0.34 and 0.42, whereas the standard deviation (s.d.) varies from 0.5 to 0.9. The mean values of MFCCs close to 0 indicate that the distribution is balanced. On the other hand, a higher standard deviation denotes the spread of diverse audio features. The pitch has a mean value of −0.06 with a standard deviation of 0.98. The standard deviation confirms the diversity in pitch similar to the standard deviations of the MFC coefficients. The mean values for spectral centroid, spectral flatness, energy, and ZCR are −0.18 with s.d. of 0.5, −0.12 with s.d. of 0.47, −0.12 with s.d. of 0.2, and −0.18 with s.d. of 0.53, respectively. Further, the data presented in Figure 7 outline the observed patterns across twenty-two features within Cluster 0. These twenty-two features contain audio features, including thirteen MFCC features (MFCC1–MFCC13), pitch, spectral centroid, spectral flatness, energy, ZCR, and spectral features such as age, gender, distance, and statements. The chart highlights that MFCC1 is the coefficient that contains mostly positive peaks, while MFCC2 to MFCC13 have both negative and positive peaks. The pitch chart contains both positive and negative peak values. The spectral centroid fluctuates between negative and positive values, while spectral flatness consistently remains positive, with a maximum peak value higher than 10. The energy pattern is predominantly positive, with a maximum peak value exceeding 25, and the Zero-Crossing Rate (ZCR) varies between positive and negative values. Regarding demographics, people aged between 25 and 30 are the highest in number in Cluster 0, and the gender distribution appears to be roughly equal, with slightly more males. The data indicate that most data points in this cluster are associated with a distance of 1 m from the robot. Furthermore, Cluster 0 shows a higher occurrence of the fourth and fifth statements compared to other statements.

The descriptive statistics of each feature of Cluster 1 are illustrated in Table 2. As per Table 2, the number of records for each feature in Cluster 1 is 12122. The mean values of MFCCs lie between −1.25 and 1.23, whereas the standard deviation (s.d.) varies from 0.96 to 1.27. The pitch has a mean value of 0.24 with a standard deviation of 1.05. The mean values for spectral centroid, spectral flatness, energy, and ZCR are −0.24 with s.d. of 0.73, −0.28 with s.d. of 0.60, 0.46 with s.d. of 2.13, and −0.17 with s.d. of 0.64, respectively. Further, Figure 8 in Cluster 1 showcases the distribution of twenty-two distinct features. These features contain audio features, including thirteen MFCC features (MFCC1–MFCC13), pitch, spectral centroid, spectral flatness, energy, and ZCR, as well as spectral features such as age, gender, distance, and statements. The graph depicts that MFCC1 has predominantly positive peaks, with the highest peak reaching greater than +5. On the other hand, other coefficients, such as MFCC2 to MFCC13, exhibit a mix of positive and negative peaks. Pitch’s highest peak value is more than 2.5, though it dips into negative values for some data records. The trend of the spectral centroid and spectral flatness varies between positive and negative peak values. Energy levels also remain mostly positive, with the highest peak exceeding 2. ZCR fluctuates between positive and negative values. Within Cluster 1, the most frequent age is 16 years, followed by individuals aged 35. Females are in a higher proportion of the cluster than males. Upon analyzing the distance from the robot, it appears that there are significantly more data records at distances of 3 and 5 m within Cluster 1.

The descriptive statistics of each feature of Cluster 2 are illustrated in Table 3. As per Table 3, the number of records for each feature in Cluster 2 is 3808. The mean values of MFCCs lie between −1.55 and 1.10, whereas the standard deviation (s.d.) varies from 0.93 to 1.30. The pitch has a mean value of −0.03 with a standard deviation of 0.98. The mean values for spectral centroid, spectral flatness, energy, and ZCR are 2.88 with s.d. of 1.51, 2.24 with s.d. of 2.59, −0.10 with s.d. of 0.30, and 2.60 with s.d. of 2.02, respectively. Moreover, Figure 9 depicts the patterns of twenty-two features within Cluster 2. The features are related to both audio features, including thirteen MFCC features (MFCC1–MFCC13), pitch, spectral centroid, spectral flatness, energy, and ZCR, as well as spectral features such as age, gender, distance, and statements. It shows that MFCC1 has mostly positive peaks, whereas other MFCCs (MFCC2 to MFCC13) display both positive and negative peaks. The trend of the spectral centroid varies, while spectral flatness remains consistently positive, with a maximum peak value of more than 50. The energy pattern is also consistently positive, with a maximum peak value exceeding 5. ZCR peaks fall between positive and negative values. Regarding the age distribution in Cluster 0, the majority falls within the 25–30 age group. In Cluster 2, there are more females than males. Analysis of the distance from the robot reveals that there are more occurrences of distances around 1 m, followed by distances of approximately 3 m. Furthermore, this cluster exhibits a higher frequency of the first and third statements.

**Figure 10:**
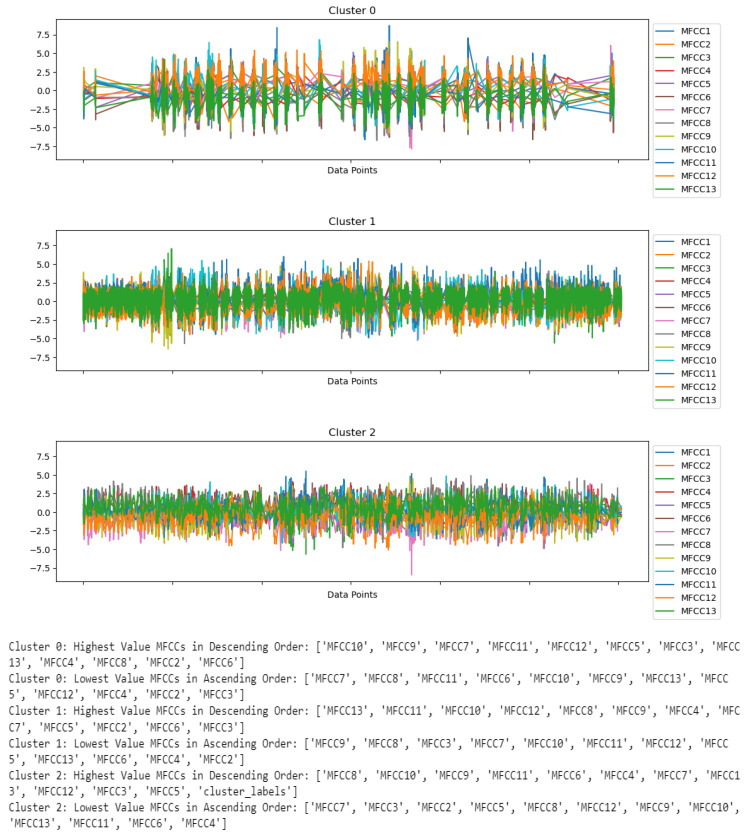
Visualization of MFCCs in each of the clusters.

Furthermore, a comprehensive examination of the MFCC features is performed, and the outcomes are illustrated in Figure 10, where MFCC features are denoted in different colors. The findings indicate that within Cluster 0, the most prominent coefficient is MFCC10, while the least significant is MFCC7. For Cluster 1, the highest coefficient is MFCC13 and the smallest is MFCC9. In contrast, within Cluster 2, MFCC8 has the highest coefficient and MFCC7 has the lowest. 

**Figure 11:**
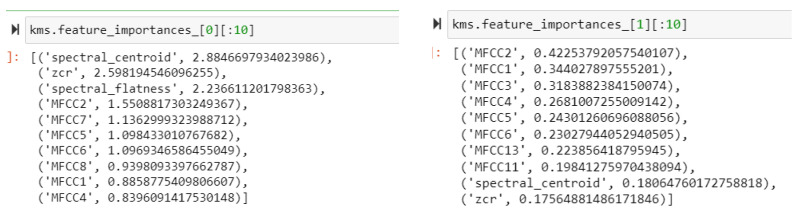

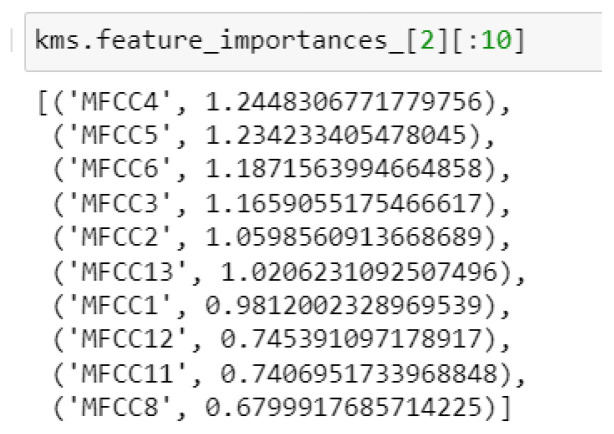
Ten most important features in three clusters.

Furthermore, the 10 most important features within each cluster were selected using the KMeansInterp function, as suggested by Yousef et al. [111]. The output obtained for the three clusters is shown in Figure 11.

### 4.3. Integration of Whisper to Evaluate Pepper’s Speech Recognition System

The recorded audio can be converted into text with the help of the Whisper speech-to-text recognition tool. The evaluation metrics suggest the extent of similarity between the Whisper-generated text and the original text. The measures WER, MER, WIL, and CER were calculated for all recordings. Then, we combined these values with the above-mentioned audio features to evaluate which cluster has the best values.

**Figure 12:**
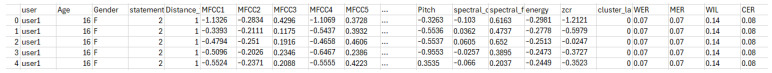
Screenshot of top 5 rows (out of 60,336 records) and 28 features, including person’s demographics, position, audio features, and evaluation metrics.

However, before the best cluster selection, imputing the missing values corresponding to WER, MER, WIL, and CER was necessary. These values were filled by taking the mean of the columns where these values were present. Figure 12 shows the 28 combined features, which include the person’s demographics, statements, position, audio features, and evaluation measures. This figure provides a glimpse of features included for analysis purposes and not the complete details of all the participants.

### 4.4. Selection of Best Cluster Depending on WER Threshold

**Figure 13:**
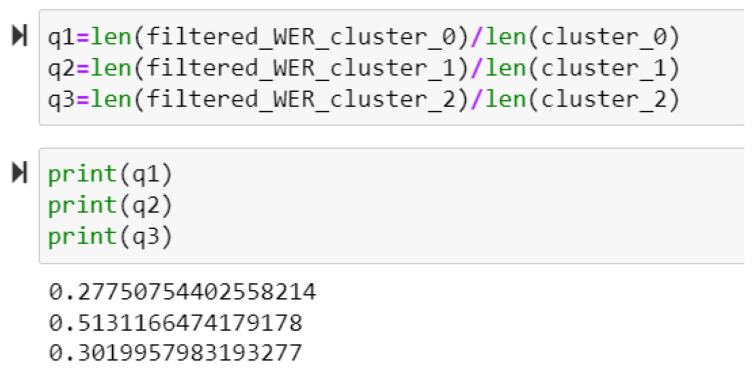
Screenshot of code snippet to select the best cluster.

The lower value of WER indicates a higher accuracy. The best cluster should contain the maximum number of WER values rather than a larger number of data points. A threshold of less than or equal to 0.3 was implemented for WER and all records were filtered. The quality of the cluster was checked, as shown in Figure 13, in which a higher value indicates the best-quality cluster. This suggests that Cluster 1 is the best cluster as it contains records with higher accuracies in speech recognition.

### 4.5. Visualization of Best Records in Cluster 1

**Figure 14:**
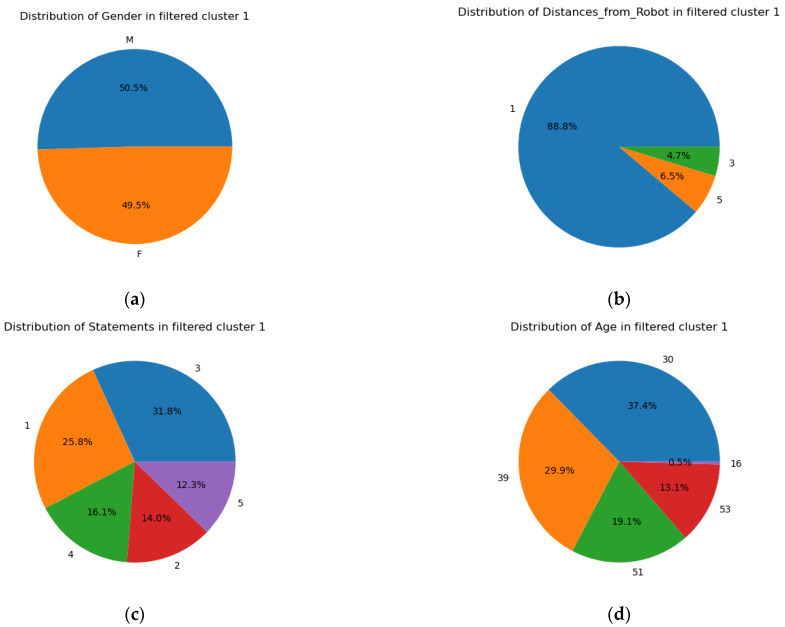
Analysis of the distribution of (**a**) gender, (**b**) distances from the robot, (**c**) statements spoken, and (**d**) age for best records in Cluster 1.

It is important to analyze the pattern of data records that have good accuracies in Cluster 1. For this purpose, different pie charts (Figure 14) have been created. Figure 14a shows the distribution of gender in filtered Cluster 1 and suggests that there is no great effect of male and female voices as they are almost equal in number. Figure 14b illustrates that speech recognition was best when the distance between the person and the robot is lowest, i.e., 1 m. At 3 m and 5 m, there is not much difference. As per Figure 14c, the most accurately recognized statement was statement3 followed by statement1. It should be noted that the statement0 was not present in this chart, which shows that that statement was not recognized. Figure 14d shows that speech recognition was best for those who are nearly 30 years of age.

## 5. Discussions

The present study employs an amalgamation of features—MFCCs, pitch, the spectral centroid, spectral flatness, energy, and the ZCR—to advance the understanding of speech recordings. These speech recordings were captured using Pepper’s speech recognition system. The objective is to understand the impact of spatial features such as age, distance from Pepper, statements, and gender on Pepper’s speech recognition system. To achieve this, the recordings were transferred to a local system for further analysis, where the unsupervised machine learning K-means technique was applied to audio features to group them based on their properties. Further, Whisper was applied for speech-to-text conversion and evaluation metrics were used to calculate the accuracy of captured audio. The cluster that contains higher accuracies was selected as the best. In the current study, more than one audio feature is selected. Previous studies [16,17,18,112] also demonstrated that the combination of features can provide comprehensive insights into speech data. For instance, Kerkeni et al. [17] proposed that combining two features can enhance the performance of speech emotion recognition (SER) performance. Similarly, Dash et al. [16] employed gradient-boosting machines to accomplish consistent performance across different datasets via the fusion of spectral, cepstral, and periodicity features. Furthermore, Jiang et al. [112] utilized a combination of MFCCs, the extended Geneva Minimalistic Acoustic Parameter Set (eGeMAPS), and high-level acoustic features from speech recognition networks to extract relevant speech-related information. Moreover, Wang et al. [18] incorporated MFCC and spectrogram features for speech emotion recognition, achieving 29.4% and 23.8% higher classification accuracy than MFCC and spectrogram features, respectively. It has been found that incorporating features such as MFCCs, the ZCR, and LPC into the speech recognition system resulted in consistent performance, even with larger numbers of participants [113]. In past studies, unsupervised learning was also applied for different purposes related to speech recognition, such as the reduction in feature space dimension [79], mood detection [81], emotion classification [82], and the classification of sound signals [83]. Similarly, in the present research, K-mean clustering is used to better understand the impact of spatial features on the speech recognition system of the Pepper robot.

In this study, relevant features were selected based on the existing literature. MFCCs, the spectral centroid, spectral flatness, and the ZCR are generally employed in speech processing to extract relevant information. Pitch and RMS are essential features for analyzing speech recordings. Davis et al. [114] suggested MFCCs as an approach to demonstrating speech’s spectral features, laying the basis for applying MFCCs as the fundamental feature set for speech analysis. Lehner et al. [115] achieved significant recognition results with a minimal and optimized feature set mostly consisting of selected MFCCs. Reports indicate that spectral centroid features effectively capture more information from subband spectral distributions, especially in speech recognition systems [116,117]. Further, spectral centroid features can also help in speech emotion detection [63,118]. Qadri et al. [119] differentiated gender-specific speech using spectral features such as the spectral centroid and spectral flatness. Another study [120] showed that spectral flatness denotes the noise-like behavior of a waveform where the high peaks indicate lower spectral flatness. The ZCR can be helpful in speech processing, including capturing information about loud and short sounds [121]. Chauhan et al. [122] proposed that integrating prosodic information with spectral features can be beneficial for advancement in speech recognition systems. Furthermore, they also suggested that pitch and RMS features are less affected by channel and noise.

There have been few studies focusing on the analysis of audio features obtained from social robots. Bird et al. [123] mentioned that Pepper and Nao robots are suitable for general speech recognition; however, they do not have the advanced capability of classifying speakers. Shen et al. [124] proposed a framework for personality trait identification through HRI using various features, including vocal features such as MFCCs, pitch, and energy. In another study [125], audio data were collected during human–robot interaction using Pepper’s built-in sensor and lapel microphone; further, MFCCs and pitch features were utilized to analyze the data. In the past, studies were conducted on the analysis of audio captured by robots other than social robots. Telembici et al. [126] extracted MFCCs to analyze the accuracy of audio captured by the service robot TIAGo. Wu et al. [127] employed a surveillance robot to identify abnormal audio sounds using MFCC feature extraction. Drawing inspiration from the above-mentioned studies, we adopted the Pepper robot for the present research.

The performance of speech recognition systems can be tested using evaluation metrics. Pande et al.’s [110] findings suggest that Whisper is the best speech recognition tool among other speech recognition tools. Furthermore, they tested the accuracy based on the evaluation measures WER, MER, WIL, and CER and found that these measures were low in the case of Whisper, which is an indicator of good performance. The continuous monitoring of these measures in the many real-world applications of Pepper can be helpful in understanding how the speech recognition system performs. This, in turn, can be advantageous for user interaction, satisfaction, and reliability. Similar to our approach in the present study, Abdelhadi et al. [128], in their research, also employed Whisper to convert audio recordings captured by the Pepper robot. 

The impact of gendered voices on speech recognition systems has been extensively explored in the field of ASR. Research studies highlight the importance of understanding and addressing gender-related differences in speech characteristics to enhance the accuracy of speech recognition technologies. These differences include variations in vocal tract length [129] and pitch [130]. Garnerin et al. [131] discovered significant differences in the performance of ASR systems, measured by the Word Error Rate (WER), between males and females. Adda-Decker et al. [132] found that females tended to achieve better speech recognition results on average for both English and French speech than males. However, a study conducted by Tatman et al. [133] suggested the opposite, finding the suitability of the male voice for speech recognition. Doddington et al. [134] discussed that both male and female voices exhibit distinct physical attributes such as pitch and vocal tract length. Furthermore, their findings suggested a minor performance difference between genders, with a slight tendency for males to perform better. The present study also demonstrated that the difference in the voice ratio between male and female participants is very small, with slightly a higher value for male voices.

The speaker’s distance from the system can affect the quality and accuracy of speech recognition. Studies by [135,136] have shown that as the speaker moves further away from the microphone, the accuracy of speech recognition decreases. Similarly, the present study shows that in best-selected Cluster 1, a shorter distance between the speaker and the microphone results in more accurate speech recognition. The existing literature also suggested an impact on vocal intensity with increasing distance [137,138]. Meyer et al. [54] examined how environmental noise and varying distances affect word recognition. Attawibulkul et al. [53] proposed that environmental noise presents a significant challenge for speech recognition in robots.

Previous research studies have demonstrated that speech recognition accuracy could be influenced by factors such as statement complexity and age. Studies [135,136] demonstrated that the minimum distance from speech recognition devices provides better and more accurate results. Our previous research [10] also indicated that the accuracy of speech recognition systems is affected by the complexity of statements, whereby simple statements made at 1 m from the Pepper robot were the most optimal. Similarly, we found in the present study that the best cluster mostly contains 1 m distance data (88.8%), suggesting speakers should maintain the minimum distance from the robot. Further, participants from different age groups were included in the experiment, and it was found that in the best-performing cluster, the majority of the participants were from the 25–30 age group. However, this could be due to the fact that our sample is skewed towards participants of that age group. The speech characteristics in older people, such as variations in the speech rate, pauses, and reduced articulation, have been identified as factors contributing to increased WERs in ASR systems [139]. Furthermore, children may also struggle with pronunciation when articulating a word. Li et al. [140] illustrated that it is comparatively more difficult for ASR to perceive children’s voices than adults. There are studies that showed problems in perceiving spoken words by social robots, including Nao and Pepper, for both children [46,48] and elders [49,50]. Educational qualification might not directly influence the speech recognition system, but it can impact various factors such as pronunciation, speaking style, and rich vocabulary, which in turn can influence the performance of the speech recognition system.

## 6. Conclusions, Implications, Limitations, and Future Work

The present study employs a humanoid robot, Pepper, to record spoken statements to evaluate its speech recognition system. Different audio features containing accurate information about the speech recordings were selected. The capability of a speech recognition system with the application of K-mean clustering on audio features can provide an effectual assessment. With reference to RQ1, the integration of the speech-to-text converter Whisper is a potential method to assess the precision of recordings performed by Pepper’s speech recognition system. With reference to RQ2, the cluster analysis of audio features suggests that the best accuracy of the speech recognition system can be found at a 1 m distance from the Pepper robot in comparison to other distances. On the other hand, age and gender do not seem to have much influence on the performance of speech recognition systems. Within a 1 m distance, the speech recognition system of Pepper performs the best to perceive the audio. Thus, it is recommended that the Pepper robot should be at a maximum distance of one meter from the speaker in diverse settings where the display of subtitles is needed along with speech.

The insights gained from the present study have a significant impact on different domains of the real world. One such domain is hospitality settings, where Pepper can be utilized as a receptionist with the facility to provide information and handle customers’ queries. Another area is healthcare, which, according to the need, Pepper, at the closest distance, can provide companionship, advice, and entertainment to different age groups, including elders and children. The proposed setup can also be advantageous for educational and meeting purposes, including online and physical classroom teaching and meetings. Furthermore, this setup could also be applied to crowded places to comprehend individuals’ speech in a better way. It not only increases the understanding of spoken words but also reduces a person’s cognitive burden. However, there are challenges in deploying Pepper in crowded places due to the presence of noise. Background noise makes it difficult for Pepper’s speech recognition system to perceive its environment efficiently. Moreover, language diversity can also pose challenges for Pepper since to support multilingual environments, switching between languages is needed, which can further impact the accuracy of the speech recognition system. Additionally, the varying accents of people and the limited vocabulary of Pepper can also create another layer of complexity for the proper comprehension of the spoken statements.

The implications of the present study have potential possibilities for advancing human–robot interactions in various contexts. Speech can play a role in the identification of a person’s emotional state, including stress and loneliness. The robot can be further programmed to provide appropriate responses after precise capturing of spoken words. The aim of these responses is to help individuals to deal with problems. The responses could be related to effective dialogue establishment. In healthcare scenarios, robots can be programmed to provide remedies and suggestions, including games and exercises, which will help to engage individuals. Meanwhile, in educational settings, the robot can be prepared to help students by providing solutions to their queries. This kind of interaction can increase their engagement and learning experience. A user-friendly robotic system can be designed in which users will have the option to select their choice for engagement; for instance, upon the identification of stress in a person’s voice by a robot, the person will have many options such as music, games, and exercises to select from the robot’s interface. Similarly, upon detection of not understanding the lecture, there will be options for students to listen to the recordings of the lecture or select another tutorial of their choice.

We experienced some difficulties with Pepper’s speech recognition system. One of the biggest problems that Pepper has is the lack of speech-to-text transcription. Therefore, the audio files need to be transferred into the local system using the Paramiko library to convert speech into text, which requires another program to run in addition to the program that is compiled for speech recording. For speech recording, the programmer had to run the same program 18 times for each participant (covering six statements at three different distances). There were 8 participants, and therefore this required running the same program 144 times, demonstrating the workload on the person collecting the data. Sometimes, during the experiment, Pepper’s in-built capabilities led it to respond to certain recognized keywords in the participant’s speech. In that case, a recording was repeated, which increased the experiment duration. Other challenges of the speech recognition system of Pepper include difficulty in understanding accents, pronunciation, and a restricted vocabulary. Background noise also poses a problem for Pepper to recognize speech accurately. The result of this study is based on a limited sample size. Further, the set of participants selected was random; however, it was skewed towards a certain group. Therefore, the findings should be further strengthened by including a bigger sample size in the future. The present study serves as a prototype for conducting the experiment in real settings. The diversity in sample collection can be maintained by incorporating participants from different countries and different age groups. It is expected that it will help in collecting diverse speech samples. The present study does not focus on emotion detection through speeches. In the future, we will also attempt to collect the emotions of participants, which are expressed through speeches, by using certain stimuli such as watching video clips or playing games.

This study is a crucial initial step in comprehending the system’s limitations in a controlled laboratory environment before implementing it in a real setting. Our findings indicate that the distance between the robot and the speaker has an impact on Pepper’s speech recognition capabilities. In the present study, we did not incorporate noise reduction techniques, which might be helpful to increase the performance of speech recognition. It should also be noted that microphones were not used for the study, and the use of microphones can further enhance the accuracy of capturing speech, even from larger distances. In future studies, we will also investigate whether the placement of the microphone with the speaker influences Pepper’s speech recognition. We plan to improve human–robot interaction and engagement by displaying the robot’s responses on its screen alongside the text.

## Figures and Tables

**Table 1:** Descriptive statistics of each feature of Cluster 0.

	MFCC1	MFCC2	MFCC3	MFCC4	MFCC5	MFCC6	MFCC7	MFCC8	MFCC9	MFCC10	MFCC11	MFCC12	MFCC13	Pitch	Spectral_Centroid	Spectral_Flatness	Energy	zcr
**count**	44,406.0	44,406.0	44,406.0	44,406.0	44,406.0	44,406.0	44,406.0	44,406.0	44,406.0	44,406.0	44,406.0	44,406.0	44,406.0	44,406.0	44,406.0	44,406.0	44,406.0	44,406.0
**mean**	−0.34	0.42	−0.32	0.27	−0.24	0.23	−0.03	0.1	−0.0	−0.12	0.2	−0.17	0.22	−0.06	−0.18	−0.12	−0.12	−0.18
**std**	0.7	0.53	0.66	0.68	0.65	0.67	0.79	0.83	0.9	0.88	0.87	0.87	0.81	0.98	0.5	0.47	0.2	0.53
**min**	−1.87	−2.28	−5.62	−3.34	−4.41	−3.42	−5.27	−5.68	−6.41	−5.11	−4.91	−4.48	−3.81	−1.21	−1.98	−0.95	−0.32	−1.83
**25%**	−0.83	0.08	−0.61	−0.16	−0.64	−0.15	−0.42	−0.33	−0.49	−0.61	−0.32	−0.7	−0.23	−0.89	−0.45	−0.45	−0.23	−0.45
**50%**	−0.57	0.45	−0.24	0.22	−0.26	0.29	0.04	0.13	0.02	−0.16	0.15	−0.2	0.28	−0.37	−0.25	−0.13	−0.2	−0.23
**75%**	−0.07	0.77	0.11	0.62	0.17	0.68	0.46	0.58	0.51	0.32	0.65	0.32	0.74	0.59	0.01	0.14	−0.08	0.04
**max**	5.23	3.34	1.96	4.06	3.60	2.96	4.00	5.26	4.77	5.52	6.02	5.40	7.07	2.57	2.95	7.76	2.32	3.87

**Table 2:** Descriptive statistics of each feature of Cluster 1.

	MFCC1	MFCC2	MFCC3	MFCC4	MFCC5	MFCC6	MFCC7	MFCC8	MFCC9	MFCC10	MFCC11	MFCC12	MFCC13	Pitch	Spectral_Centroid	Spectral_Flatness	Energy	zcr
**count**	12,122.00	12,122.00	12,122.00	12,122.00	12,122.00	12,122.00	12,122.00	12,122.00	12,122.00	12,122.00	12,122.00	12,122.00	12,122.00	12,122.00	12,122.00	12,122.00	12,122.00	12,122.00
**mean**	0.98	−1.06	1.17	−1.25	1.23	−1.19	0.46	−0.68	0.15	0.34	−0.74	0.75	−1.02	0.24	−0.24	−0.28	0.46	−0.17
**std**	1.12	1.06	1.05	0.96	1.01	0.97	1.27	1.08	1.18	1.24	1.08	1.08	0.94	1.05	0.73	0.60	2.13	0.64
**min**	−1.16	−4.21	−3.70	−4.45	−4.62	−6.60	−7.85	−6.72	−6.05	−6.21	−6.61	−4.50	−5.23	−1.21	−2.38	−0.96	−0.30	−1.76
**25%**	0.07	−1.50	0.66	−1.80	0.96	−1.44	0.12	−1.11	−0.42	−0.29	−1.39	0.15	−1.53	−0.73	−0.64	−0.76	−0.21	−0.54
**50%**	0.69	−1.17	1.43	−1.45	1.40	−1.03	0.81	−0.48	0.14	0.38	−0.83	0.73	−0.94	0.17	−0.30	−0.35	−0.09	−0.23
**75%**	1.61	−0.59	1.79	−0.84	1.75	−0.71	1.19	−0.03	0.74	0.98	−0.21	1.33	−0.45	1.21	0.05	0.05	0.33	0.08
**max**	8.72	3.15	4.38	3.65	5.13	1.95	6.05	3.36	6.56	6.82	5.56	5.46	4.20	2.57	2.91	10.58	35.54	4.11

**Table 3:** Descriptive statistics of each feature of Cluster 2.

	MFCC1	MFCC2	MFCC3	MFCC4	MFCC5	MFCC6	MFCC7	MFCC8	MFCC9	MFCC10	MFCC11	MFCC12	MFCC13	Pitch	Spectral_Centroid	Spectral_Flatness	Energy	zcr
**count**	3808.00	3808.00	3808.00	3808.00	3808.00	3808.00	3808.00	3808.00	3808.00	3808.00	3808.00	3808.00	3808.00	3808.00	3808.00	3808.00	3808.00	3808.00
**mean**	0.89	−1.55	−0.00	0.84	−1.10	1.10	−1.14	0.94	−0.48	0.37	0.05	−0.37	0.64	−0.03	2.88	2.24	−0.10	2.60
**std**	0.93	1.00	1.30	1.05	0.97	1.14	1.25	1.27	1.30	1.07	1.04	1.02	1.04	0.98	1.51	2.59	0.30	2.02
**min**	−1.08	−5.09	−5.67	−2.00	−4.90	−3.19	−8.44	−4.79	−4.18	−4.17	−3.60	−4.44	−3.77	−1.21	−0.45	−0.94	−0.31	−1.54
**25%**	0.20	−2.17	−0.70	0.11	−1.75	0.42	−2.04	0.09	−1.40	−0.35	−0.64	−1.03	−0.01	−0.89	1.81	1.11	−0.21	1.24
**50%**	0.74	−1.34	0.17	0.77	−1.20	1.21	−1.18	0.92	−0.48	0.37	0.02	−0.40	0.71	−0.36	2.50	1.83	−0.18	2.12
**75%**	1.40	−0.82	0.82	1.56	−0.47	1.93	−0.26	1.81	0.39	1.08	0.70	0.27	1.34	0.82	3.62	2.83	−0.09	3.44
**max**	5.49	0.55	3.20	4.07	2.48	4.21	3.82	4.90	4.68	4.84	4.48	3.44	3.50	2.57	9.77	58.17	6.64	14.53

## Data Availability

Data will be provided upon request.
